# Translating extracellular vesicle packaging into therapeutic applications

**DOI:** 10.3389/fimmu.2022.946422

**Published:** 2022-08-15

**Authors:** Dilara C. Ozkocak, Thanh Kha Phan, Ivan K. H. Poon

**Affiliations:** Department of Biochemistry and Chemistry, La Trobe Institute for Molecular Science, La Trobe University, Melbourne, VIC, Australia

**Keywords:** extracellular vesicles, cargo packaging, EV therapies, drug delivery, EV biogenesis

## Abstract

Extracellular vesicles (EVs) are membrane-bound particles released by cells in various (patho)physiological conditions. EVs can transfer effector molecules and elicit potent responses in recipient cells, making them attractive therapeutic agents and drug delivery platforms. In contrast to their tremendous potential, only a few EV-based therapies and drug delivery have been approved for clinical use, which is largely attributed to limited therapeutic loading technologies and efficiency. As EV cargo has major influence on their functionality, understanding and translating the biology underlying the packaging and transferring of biomolecule cargos (e.g. miRNAs, pathogen antigens, small molecule drugs) into EVs is key in harnessing their therapeutic potential. In this review, through recent insights into EVs’ content packaging, we discuss different mechanisms utilized by EVs during cargo packaging, and how one might therapeutically exploit this process. Apart from the well-characterized EVs like exosomes and microvesicles, we also cover the less-studied and other EV subtypes like apoptotic bodies, large oncosomes, bacterial outer membrane vesicles, and migrasomes to highlight therapeutically-diverse opportunities of EV armoury.

## Introduction

**Figure 1:**
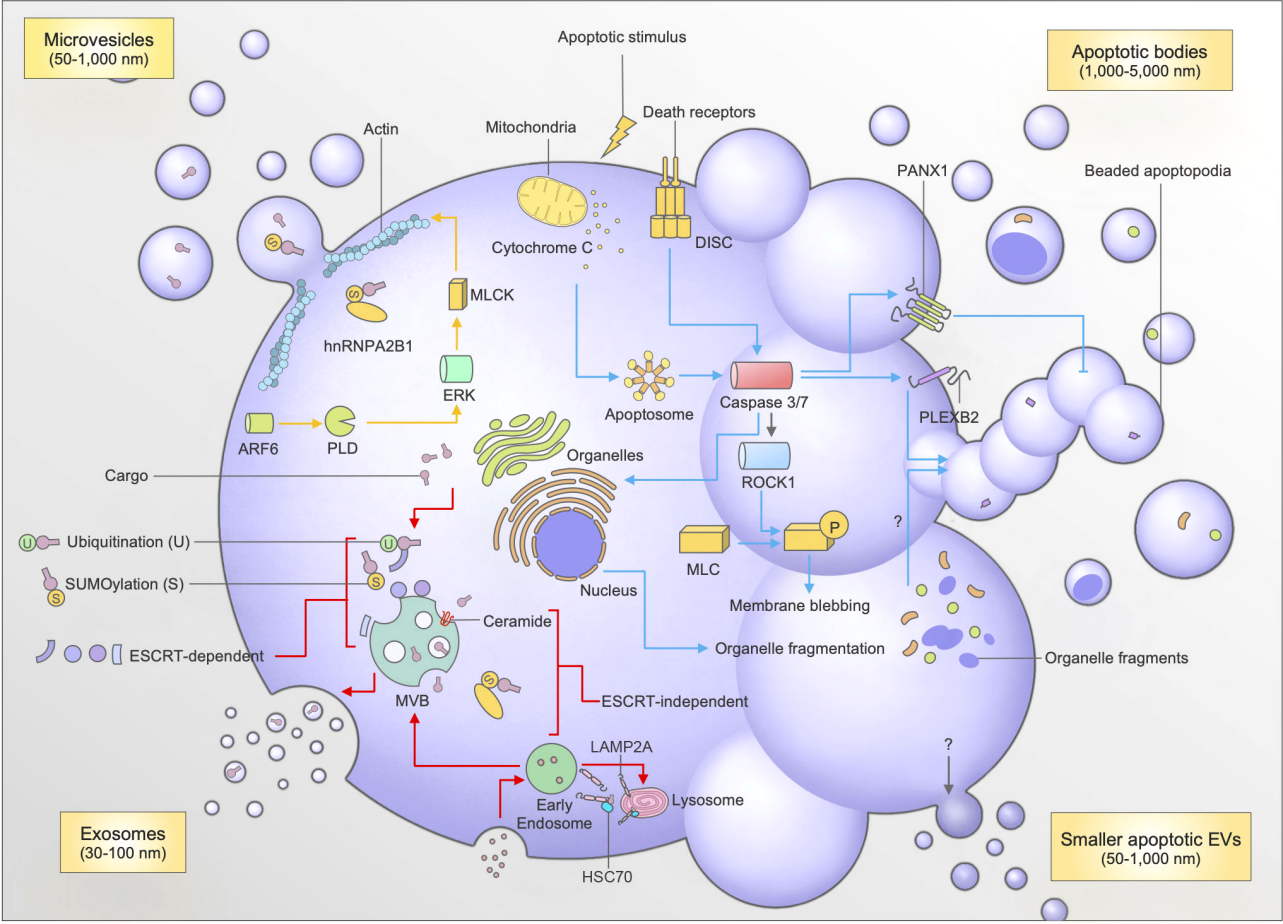
Mechanisms of biogenesis and cargo packaging for exosomes, microvesicles (MVs), apoptotic bodies (ApoBDs), and small apoptotic EVs. Exosome biogenesis (red arrows) involves the formation of intraluminal vesicles (ILVs) which contain cargo trafficked to the multivesicular body (MVB). The trafficking of cargo to the MVB involves post-translational modifications like sumoylation and ubiquitination by proteins like hnRNPA2B1, and interaction between ESCRT machinery (ESCRT-dependent), or sphingolipid ceramide, and LAMP2A-HSC70 complexes (ESCRT-independent). The fusion of the MVB with the plasma membrane causes the release of exosomes into the extracellular milleu. The formation of microvesicles (yellow arrows) occurs through plasma membrane budding, which requires actomyosin contractions facilitated by ARF6. Apoptotic bodies are generated following an apoptotic stimulus (blue arrows), which facilitates the induction of the extrinsic (death receptor mediated) or intrinsic (mitochondrial) pathway of apoptosis. The subsequent formation of the Apoptosome or DISC activates caspases 3 and 7. Caspases 3 and 7 cleave and activate PANX1 (negative regulator of apoptotic cell disassembly), ROCK1 (to facilitate membrane blebbing), and PLEXB2 (regulator of beaded apoptopodia formation). Caspases 3 and 7 are also able to cleave proteins to aid organellar fragmentation, which can then subsequently be packaged into ApoBDs through an unknown mechanism. Apoptotic EVs are also released during apoptosis, however the mechanisms are currently unknown.

At any given moment cells constitutively release signals enabling them to communicate with other cells. Among these signals are small, heterogenous populations of membrane-bound vesicles known as extracellular vesicles (EVs). EVs are traditionally classed into 3 major categories, which are exosomes, microvesicles (MVs), and apoptotic bodies (ApoBDs) **(** **Figure 1** **)**. However, the EV field has since expanded considerably to include the emergence other EV subtypes (1). They are released under varying conditions such as in the case of cell transformation, cell migration and other forms of programmed cell death like necroptosis (2–5). EVs can also be released by microbes such as in the case of bacterial outer-membrane vesicles (OMVs), fungal EVs and parasitic EVs (6–8).

Through transfer of functionally active biomolecules such as proteins and nucleic acids, EVs act as important mediators for intercellular communication in multiple physiological and pathological settings. Notably, EV amounts and cargo can be dysregulated during and contribute to the progression of infections, cancer, and neurodegeneration (9–12). As a consequence, EVs are considered attractive targets for novel therapeutic designs, which is why a number of EV-targeting approaches have been newly devised to control the formation and contents of endogenously formed EVs (13–15). Additionally, EVs display inherently clinically-desirable characteristics for therapeutic use such as (i) the ability to contain diverse biomolecular cargos, (ii) the ability of said cargo to elicit potent cellular responses, (iii) the ability to cross biological barriers, (iii) availability, (iv) bioengineerability and (v) scalability (10, 16–18). Furthermore, EVs have been shown to be safe for clinical use, for instance, loading EVs with a common chemotherapeutic drug, doxorubicin, was able to increase efficacy of the drug whilst simultaneously significantly reducing side effects in patients (11, 19). Other pre-clinical and clinical trials have also demonstrated the safety of mesenchymal stem cell (MSC) derived-EVs, citing low immunogenicity, although this is currently still an active area of investigation (see Janockova et al., 2021 for an in-depth review) (20, 21). Additionally, the malleability of EVs has provided opportunities for ingenuity, leading to favorable treatment outcomes for patients undergoing anticancer therapy, pathogen vaccination, immunotherapy and regenerative therapies (11, 12, 22–26). Innovative approaches such as formation of synthetic vesicles as cancer immunotherapies, as well as chimeric apoptotic vesicles have provided strong promise for such use (27, 28). Many groups have also investigated the use of EVs like exosomes, MVs, ApoBDs and large oncosomes (LOs) in minimally-invasive diagnostic strategies (26, 29–32). More recently, the approach of using EVs to help vaccinate and protect against SARS-CoV-2 infection is being investigated (33, 34). Nevertheless, the development of EV-based therapies and diagnostics is a fast-growing and promising research area.

The particular importance of EV contents in EV functions, disease progression and for therapeutic success emphasizes that understanding the biology underlying the trafficking and packaging of biomolecule cargos into EVs is key to fully unleash their therapeutic potential. In fact, EV researchers have acquired a substantial amount of knowledge to uncover the biology of cargo packaging over the past few years. Intriguingly, each EV subtype reportedly undergoes distinct cargo sorting mechanisms, implying subtype-specific functionality and therapeutic applications and collectively furthering EV diversity. Because recent EV-focused reviews have overlooked this significant feature of EV biology and had a tendency to focus on the traditional subtypes (9, 10, 12, 35), we herein capture recent advances in understanding the biogenesis of various EV subtypes, and in particular discuss the mechanisms behind cargo sorting into EVs. Furthermore, we aim to discuss the possible strategies available to utilize these EV packaging mechanisms for the purpose of developing a diverse repertoire of EV-based therapeutics.

## Mechanisms of cargo sorting into extracellular vesicles

### Exosomes

Exosomes are perhaps the most well-characterized type of EV following their initial discovery in reticulocytes in 1987 (1). Ranging from 30-100 nm in diameter, exosomes originate as intraluminal vesicles (ILVs), which are formed through budding of the multivesicular body (MVB) (26). The fusion of the MVB with the plasma membrane causes the release of exosomes into the extracellular environment **(** **Figure 1** **)**. During the formation of the MVB, many factors contribute to the biogenesis and subsequent packaging of cargo into exosomes. Additionally, the identification of certain exosomal markers has helped to identify the specific mechanisms behind biogenesis and packaging. For instance, the identification of tetraspanins CD63, Alix, TSG101 enrichment in exosomes has helped in elucidating an ‘endosomal sorting complexes required for transport (ESCRT)-dependent’ pathway of exosome biogenesis (26, 36).

An interesting aspect of these pathways in exosome biogenesis is that they can interact with other accessory proteins to assist with packaging of exosomal cargo. Recent work has uncovered the importance of post-translational modifications (PTM) to biomolecules in facilitating interactions with accessory molecules to chaperone biomolecules to the MVB for packaging into exosomes (37, 38). One of the primary signals associated with protein sorting into exosomes is ubiquitination **(** **Figure 1** **)**. Ubiquitination requires the addition of one (mono-) or multiple (poly-) ubiquitin molecules onto lysine residues in protein cargo (37, 38). Following this process, ubiquitinated proteins can associate with ESCRT members ESCRT-0 and -1 by interacting with their specific proteins known as Hrs and TSG101, respectively (26, 39, 40). The clustering of ubiquitinated proteins to ESCRT-0 and subsequent recruitment to the endosomal membrane by other ESCRT members through interaction with membrane phosphoinositol PI3P ensures the sorting of specific protein cargo into exosomes during their biogenesis (26, 38–40). For instance, ubiquitination is important for transporting soluble *Mycobacterium tuberculosis* proteins into exosomes (39). Interestingly, this can have therapeutic implications as mice vaccinated with a combination of the BCG vaccine and macrophage-derived exosomes containing *M. tuberculosis* proteins were protected against tuberculosis challenge (41).

Another PTM like ubiquitination called sumoylation has also been shown to assist with cargo packaging during exosome biogenesis **(** **Figure 1** **)**. Sumoylation involves the conjugation of a small ubiquitin-like modifier (SUMO) to a protein of interest, thereby targeting it for trafficking to MVBs during vesicle formation (37, 38). This process has been shown to be paramount in facilitating trafficking of miRNAs into exosomes, through the binding of sumoylated RNA binding protein hnRNPA2B1 to specific targeting motifs on miRNAs of interest (42). Sumoylation can also assist with trafficking α-synuclein particles to small EVs through interactions with ESCRT machinery TSG101, and accessory tetraspanin Alix (43). Additionally, the associations of proteins with tetraspanin-enriched microdomains (TEMs), which are responsible for the packaging of certain cargo like MHC II into dendritic cell (DC)-derived exosomes, has been shown to be regulated by palmitoylation, which is another form of PTM (37).

To add further complexities to this pathway, cargo-sorting into exosomes can occur independent of ESCRT machinery as well. In fact, the identification of alternative exosomal sorting pathways is a subject continually under investigation. Groups have discovered the role of sphingolipid ceramide (enriched on exosomal membranes) in trafficking proteolipid proteins in murine oligodendrogial cells (44). Other recent work continues to unveil additional mechanisms utilizing other proteins to traffic specific cargo such as toll-like receptor trafficking protein UNC93B1, a syndecan-syntenin-Alix pathway, and more recently a joint pathway primarily involving LAMP2A and HSC70 to chaperone proteins like HIF1A by targeting KFERQ-like motifs (44, 45). This no doubt provides vast potential for use in EV-based therapeutics by offering alternative targets for compound loading (37, 38, 45). It also should be noted that other EV subtypes display their own selective and unique mechanisms of cargo packaging, which are described further below **(** **Table 1** **)**.

**Table 1:** Comparison between different extracellular vesicle subtypes.

EV	Size	Types of cells released by	Biogenesis	Markers	Packaging mechanisms	Cargo
Exosome	30-150 nm	Viable cells	ESCRT-dependent and independent pathways	CD63, CD9, Alix, TSG101, HSP70	Sorting based on post-translational modifications (ubiquitination, sumoylation by hnRNPA2B1, glycosylation), associations with TEMs.	Proteins, DNA, RNA, lipids
Microvesicle	50-1,000 nm	Viable cells	Budding from plasma membrane	Phosphatidylserine, Flotillin-2, selectin, integrin, CD40	ARF6 mediated selection, SNARE protein interactions, hnRNPA2B1 for the packaging of miRNAs.	Proteins, DNA, RNA, lipids
Apoptotic bodies	1,000-5,000 nm	Apoptotic cells	Budding from plasma membrane during apoptosis	Not properly defined. Phosphatidylserine exposure in conjunction with other morphological changes are used to differentiate between other particles.	Unknown	Proteins, DNA, RNA, lipids, organelle fragments, fragments of membrane protrusions
Small apoptotic EVs	50-1,000 nm	Apoptotic cells	Unknown	Not properly defined. However synthenin, and 20S Proteosome α3 have been proposed.	Unknown	Proteins
Migrasomes	500-3,000 nm	Migratory cells	Breakage of retraction fibres during migration	TSPAN4, TSPAN7, integrin-β1	Unknown	Proteins
Large oncosomes	1,000-10,000 nm	Prostate cancer cells, potentially other tumor-derived cells	Budding from plasma membrane during non-apoptotic blebbing	Cytokeratin-18, Cav-1	Unknown, thought to be cancer-specific.	Proteins, DNA, RNA, lipids
Bacterial OMVs	20-150 nm	Gram-positive and negative bacterial cells	Generation occurs in response to either: i) changes in lippoprotein crosslinks, ii) accumulation of periplasmic cargo, iii) increased membrane curvature	Bacterial species specific membrane markers	Differs according to bacterial species. LPS mediated or charge-based packaging mechanisms have been suggested in some bacterial species.	Proteins, DNA, RNA, lipids

### Microvesicles

MVs are another major category of EVs released by cells. They are generated through the budding of the plasma membrane primarily from healthy cells, and typically range from 50-1,000 nm in diameter. The biogenesis of MVs, although not as well characterized as exosomes, relies on specific cytoskeletal rearrangements and phospholipid redistributions (36, 46, 47). For membrane budding to occur, ADP-ribosylation factor (ARF6) activates phospholipase D (PLD) (48). Next, ERK is recruited to the plasma membrane to initiate phosphorylation of myosin light chain kinase (MLCK) (48). This initiates an actomyosin contraction, which then triggers the ‘pinching’ and release of the MVs from the plasma membrane into the extracellular milieu **(** **Figure 1** **) (** 48). Other external factors may also facilitate MV release, like calcium influx to induce phospholipid redistribution, indicated by PS exposure on the outer membrane leaflet (36, 46, 47). Additionally, in hypoxic conditions, ARF1 and small GTPases like RhoA and Rab22A are known to facilitate vesicle budding through actomyosin contractions through a Rho associated protein kinase (ROCK) mediated pathway (48, 49). Interestingly, although not well characterized, these pathways may bare similarities in the generation of other membrane-derived EVs including ApoBDs or large oncosomes **(** **Table 1** **)**.

MVs have displayed multiple selective mechanisms for the assortment of cargo during biogenesis (50). ARF6, which is involved in MV biogenesis, is also a key mediator for cargo selection. For instance, ARF6 is responsible for the packaging of vesicle associated membrane protein (VAMP3), integrin β-1, and MHC I into tumor-cell derived MVs, as well as the simultaneous exclusion of transferrin receptors (46, 48, 50). Furthermore, recently an ARF6-exportin 5 dependent pathway was described for the packaging of miRNAs into tumor-derived MVs (51). Other packaging mechanisms also involve the use of SNARE proteins, particularly VAMP3, which when associated with CD9 facilitates its packaging into tumor-derived MVs (36, 52). Additionally, caveolin-1, which is a MV membrane protein marker, is known to interact with hnRNPA2B1 in noxious conditions to facilitate transfer of miR-17, 93 and 20a into MVs (53).

### Apoptotic bodies (ApoBDs)

In contrast to other EVs, ApoBDs are released strictly by cells undergoing a form of programmed cell death called apoptosis. Initially thought to be generated *via* a stochastic process, recent work has revealed a highly coordinated mechanism of ApoBD formation *via* a process known as apoptotic cell disassembly (54, 55). This process details the morphological steps required for an apoptotic cell to fragment into ApoBDs, which could aid the efficient clearance of apoptotic materials (56). Previous studies have described a number of molecular regulators of ApoBD formation, namely ROCK1, Pannexin-1 (PANX1), and Plexin-B2 (PLEXB2) **(** **Figure 1** **) (** 55, 57–59). ROCK1 was found to promote apoptotic cell removal by phagocytes through controlling membrane blebbing, which is a key morphological step in ApoBD formation for certain cell types (57, 60). Furthermore, targeting the key negative regulator of apoptotic cell disassembly, PANX1, induced generation of thin string-like membrane protrusions known as apoptopodia, which promoted ApoBD formation and subsequent uptake by phagocytes (55, 58). More recently, PLEXB2 was demonstrated to regulate the generation of monocyte-derived ApoBDs, which was also important in aiding apoptotic monocyte clearance *in vitro* and *in vivo (* 59).

To further assist cell clearance, ApoBDs can contain organellar constituents that could aid recognition by immune surveillance mechanisms. For instance, the release of nuclear material like histones from membrane lysed ApoBDs could aid the recruitment of macrophages to apoptotic cells **(** **Table 1** **) (** 56, 60). Similarly, the exposure of ER proteins, ERp57 and calreticulin, could promote the immunogenicity and clearance of apoptotic material (61). Furthermore, fragments of the nucleus, cis-Golgi, mitochondria, and lysosome have also been found in ApoBDs (58, 62, 63). Although the exact functional consequences of sorting organellar fragments into ApoBDs is currently unknown, evidence suggests that this process could assist with preferential clearance of apoptotic material (64). Notably, the removal of nuclear contents from apoptotic cells is especially important in preventing inflammation due to autoantigen production, as antibodies toward nuclear autoantigens appear in high titres in many conditions including the autoimmune disease systemic lupus erythematosus (SLE) (65, 66).

Besides packaging cargo into ApoBDs for clearance and degradation, ApoBDs have also been shown to transfer specific cargo to facilitate intercellular communication **(** **Table 1** **)**. Like other EVs, ApoBDs are known to contain nucleic acids such as DNA and miRNA (17, 54). One of the initial studies on ApoBD cargo detailed the horizontal transfer of both genomic and EBV-DNA to recipient cells (67). A follow up study by the same group also demonstrated that oncogenes H-RASV12 and c-Myc could be transferred *via* ApoBDs to recipient p53^-/-^ cells, thereby promoting a tumorigenic phenotype (68). Although these studies did not specifically isolate ApoBDs, it provided the basis for many studies into ApoBD packaging and function. More recently, *in vivo* studies showed the carriage of Wnt8a particles in ApoBDs could enhance stem cell proliferation in zebrafish (24). Although it was proposed that ApoBDs could only transfer molecules to benefit the recipient cell (68), it is still unclear whether ApoBD contents are packaged passively or selectively during apoptosis. For instance, exposure and enrichment of apoptotic membrane markers (like PS) and cell-type specific markers on ApoBDs is likely to be acquired passively as these molecules are readily present at the plasma membrane (63). Additionally, although there is debate in the field, there are currently no known specific ApoBD membrane markers, which would typically help with elucidating packaging mechanisms (as mentioned for exosomes and MVs above) (69). For the acquisition or exclusion of organelle-derived content, it is slightly more complex. In packaging nuclear material, it is known that the ‘tearing apart’ action facilitated by membrane blebbing is key in aiding the partition of nuclear fragments into blebs and subsequently ApoBDs (60, 62). This is further exemplified by the exclusion of nuclear material from ApoBDs generated by THP-1 monocytes, which do not readily undergo a dynamic blebbing process (17, 58). Furthermore, as the fragmentation of the Golgi due to caspase-dependent cleavage of structural proteins during apoptosis is well documented, it is unclear whether such Golgi fragments could be shuttled in ApoBDs selectively through a distinct mechanism, or passively packaged into ApoBDs as Golgi fragments are dispersed in the cytosol (70, 71). Notably, further studies into ApoBD content have unveiled less conventional biomolecules, like the transfer of Influenza A virions in monocyte-derived ApoBDs to promote viral dissemination *via* a ‘trojan-horse’ mechanism (72). Whilst these mechanisms are complex, and vastly contrasted to other EV subtypes, there is no doubt that ApoBD packaging mechanisms display intriguing potential for exploitation in therapeutics.

Besides ApoBDs, small EVs can also be released during apoptosis, namely small apoptotic-derived EVs (**Figure 1** **) (** 73). Interestingly, Schiller et al. (2008) demonstrated that small apoptotic-derived EVs (~500 nm) could selectively carry histone proteins whilst excluding cytochrome C, prohibitin, HSP70 and lamin B (64). Likewise, small apoptotic EVs can carry a host of other effector molecules including Sjögren’s syndrome-associated autoantigen α-fodrin and 20S proteosome complexes (73–75). More recent studies into MSC-derived apoVs have shown they harbor the capability to contain multitudes of proteins and carry specific ligands like Fas-L to promote wound healing and attenuate sepsis (76, 77). However, it must be noted that whilst groups have isolated and analyzed these apoptotic-derived EVs, their characteristics such as markers, size, and method of isolation remains to be fully defined (69). Additionally, although there is evidence for selective packaging of cellular contents into apoptotic-derived EVs, their exact mechanisms of packaging and biogenesis have also not been fully elucidated. Similarly, small EVs released under other cell death conditions have also been described including pyroptosis and necroptosis (2, 78–80). Although they have only recently been described, further investigations into their contents and packaging mechanisms may provide broader treatment options and further expand treatment for inflammatory disease conditions that pyroptosis and necroptosis are associated with.

### Other EV subtypes

Over the years, investigation into exosomes, MVs and ApoBDs have demonstrated the possession of unique mechanisms required for the packaging of effector molecules. As the EV field expands to include newly characterized EV subtypes released under different conditions, it is of utmost importance to investigate their cargo and packaging mechanisms to further understand the intricacies of EV biology. This is essential, as biomolecule carriage and transfer is closely linked to function. Importantly, further understanding these mechanisms may provide insight into the causation, and treatment of patho(physiological) conditions. Here, we review other EV subtypes and recent advances that provide insight into packaging mechanisms and address their potential therapeutic use (**Table 1**).

#### Bacterial outer membrane vesicles (OMVs)

**Figure 2:**
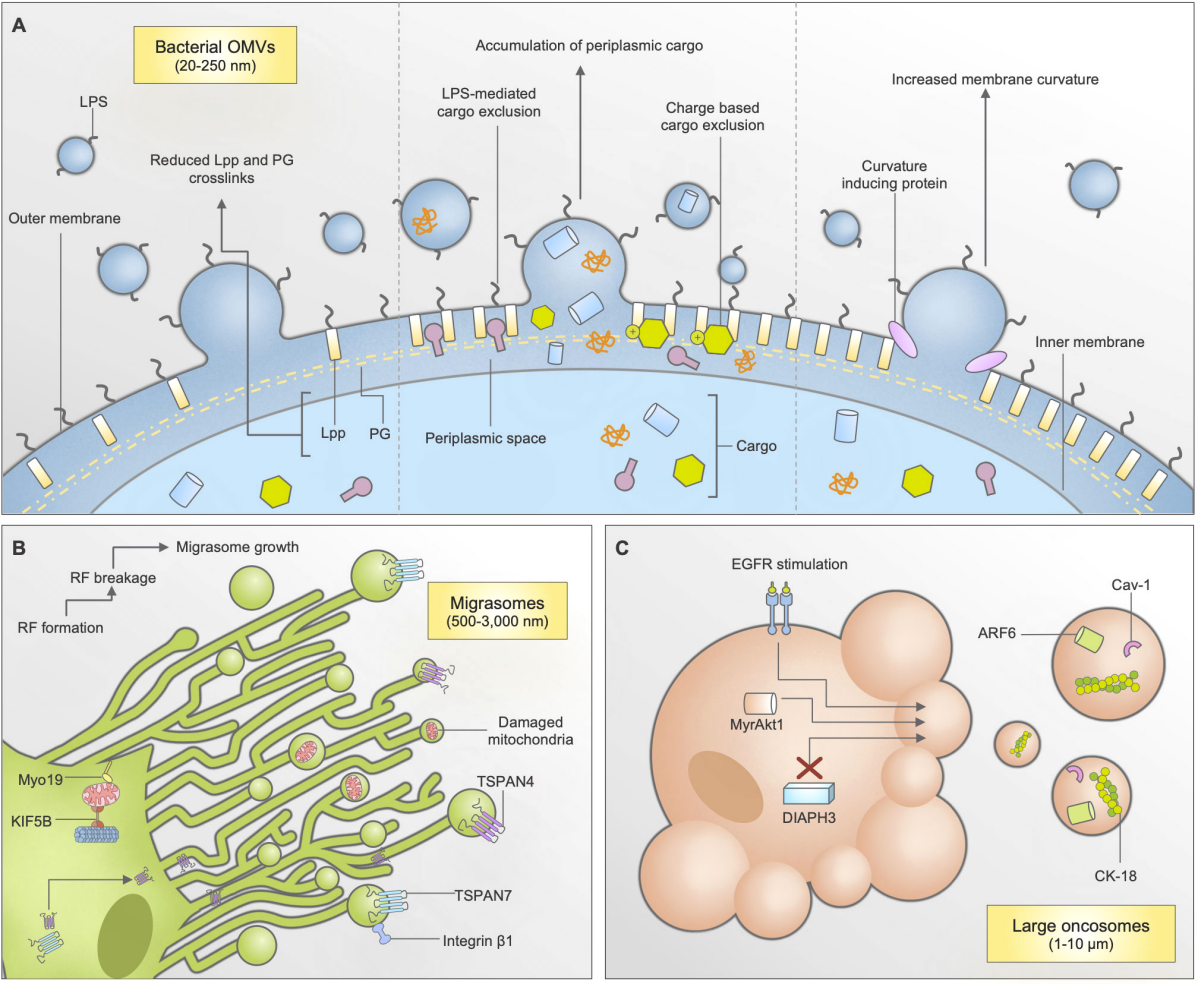
Biogenesis and cargo packaging mechanisms of bacterial outer membrane vesicles (OMVs), migrasomes, and large oncosomes (LOs). **(A)** Bacterial OMVs can be released through multiple mechanisms: through reduction in lipoprotein (lpp) and peptidoglycan (PG) crosslinks, accumulation of cargo within the periplasmic space, and increases in membrane curvature due to curvature inducing proteins. Cargo packaging mechanisms are largely unknown, however some bacterial species have indicated charge-based and lipopolysaccharide (LPS) mediated mechanisms. **(B)** Migrasomes are formed through a migration-dependent mechanisms, predominantly found in epithelial cells. As cells migrate, retraction fibres (RFs) form on the extracellular matrix. As the cell continues to move, the RF breaks and begins to form small, bulbous structures known as migrasomes. Tetraspanins TSPAN4 and TSPAN7 are known to be important for this process, and are recruited through the retraction fibres. When mitochondrial stress occurs, cells can also shuttle damaged mitochondria for disposal using mitocytosis, dependent on KIF5B and Myo19. **(C)** Large oncosomes are generated by prostate cancer cells during a non-apoptotic form of membrane blebbing. This occurs following EGFR stimulation, DIAPH3 silencing, or by activation of MyrAkt1.

Bacterial OMVs are a type of EV released by both gram-negative and gram-positive strains of bacteria. Initially observed during *Vibrio cholerae* growth, OMVs were thought to be cell debris or microscopic artefacts due to their small size of 20-250 nm (81). Since then, research has demonstrated that OMVs are extremely important to bacterial pathogenesis, as they are key in aiding the transferral of virulence factors, DNA, and contributing to biofilm formation (82, 83). With perhaps a more diverse mechanism of biogenesis among EV subtypes, OMV biogenesis can occur through a myriad of mechanisms, including changes in lipoprotein Lpp and peptidoglycan (PG) crosslinks within the membrane, accumulation of periplasmic cargo, and increase in membrane curvature (6) **(** **Figure 2A** **)**. Notably, the mechanism of OMV biogenesis may vary depending on bacterial species. It is well known that OMVs, like other EVs, can contain many parental cell-derived constituents. This includes periplasmic and cytoplasmic proteins, lipopolysaccharide (LPS), PG, DNA, RNA and enzymes (81, 84). Interestingly, certain bacterial species have demonstrated selective packaging properties for the movement of cargo into OMVs **(** **Table 1** **)**. Although, it must be noted that the exact machinery behind packaging has remained elusive. For instance, *Porphyromonas gingivalis* was able to specifically package a major group of virulence factors known as gingipans into OMVs through an unspecified LPS-mediated mechanism (82). Furthermore, it was found that LPS was also important for the exclusion of outer membrane proteins like RagA/B, further exemplifying the selectivity of this process (82). Other species of bacteria like *Bacteroides fragilis* and *Bacteroides thetaiotaomicron* could, through unspecified molecular machinery, select proteins for OMV packaging based on charge, as a majority of the cargo enriched in OMVs were acidic proteases and glycosidases (85). Notably, *Helicobacter pylori* also displayed undetermined selection mechanisms, as OMVs derived from *H. pylori* contained most T4SS components except VirD4 (6, 86). Interestingly, it has been suggested that exclusion of VirD4 from OMVs could benefit the parental bacterium (6, 86). Proteomic analysis demonstrated that OMVs from *Neisseria meningitidis* were highly enriched with 5 out of the 6 known autotransporter proteins (6, 87). Although OMV-based vaccines have been approved for use in the protection against meningococcal B, further investigations into the machinery behind bacterial OMV packaging may provide further opportunities for vaccine-based approaches. This is currently under investigation particularly in fight against COVID-19 infection (33).

#### Migrasomes

Migrasomes are a newly discovered type of EV. Ranging from 500-3,000 nm in diameter, they are formed through a unique, migration-dependent mechanism whereby the breakage of retraction fibres left behind by migrating cells begin to form small, bulbous structures over time **(** **Figure 2B** **) (** 4). The formation of migrasomes is dependent on ROCK1, tetraspanins TSPAN4, TSPAN7, integrins α5, β1, and cholesterol, in which TSPAN4, TSPAN7, and integrin-β1 are primarily used as membrane markers for migrasome isolation (88–91). Interestingly, migrasomes are formed by a broad range of cell types and have been observed in mice and zebrafish tissue (4, 88). Notably, their role in zebrafish gastrulation revealed that migrasomes, like other EVs, are enriched in many cellular signaling factors including chemokines, growth factors and morphogens (88). Intriguingly, fluorescence recovery after photobleaching (FRAP) studies have suggested that migrasomes are able to quickly recruit cellular contents as demonstrated by rapid movement of TSPAN4 into migrasomal membranes during formation, by using retraction fibres as a path (90). More recently, studies have revealed a new cargo-packaging method that neutrophils can utilize migrasomes as “disposal” systems, by specifically shuttling damaged mitochondria into them as part of a mitochondrial quality control process (92). In comparison to cargo recruitment mechanisms displayed by other EVs, these mechanisms are vastly unique and suggests a rapid and specific intracellular mechanism of recruitment, harness able for therapeutic usage. Additionally, the enrichment of enzyme proteins like N-sulfotransferase-1 (NDST1) and carboxypeptidase (CPQ), and simultaneous exclusion of organelle-derived proteins like Sec61a and GM130 suggests a selective process of packaging, although this has not been further investigated (93). The disposal of “faulty” cargo into migrasomes (whether it be proteins or organelle-specific) remains to be further investigated.

#### Large oncosomes (LOs)

LOs are the largest subtype of EVs formed specifically by tumor cells, at 1-10 μm in diameter (94, 95). Their biogenesis is associated with a non-apoptotic form of plasma membrane blebbing that occurs during cell transition to an ‘amoeboid’ phenotype **(** **Figure 2C** **) (** 95, 96). LO formation is enhanced through a variety of ways, including DIAPH3 silencing, epidermal growth factor (EGF) stimulation, and induced expression of oncoproteins like MyrAkt1 (95). LOs are distinct from exosomes in their size, and that they carry negligible levels of exosomal markers like CD81, TSG101 and CD9 **(** **Table 1** **) (** 3). Furthermore, they are known to carry a substantial amount of DNA compared to smaller EVs, which can assist in understanding cancer-specific genomic alterations (94). Interestingly, they bare a resemblance to MVs through their enrichment of ARF6 and Cav-1 (97). Another protein marker important for LO isolation is cytokeratin-18, which can be used to visualize LOs in human tissues through immunohistological studies (3).

Unlike other EVs, the majority of LO studies has been in the context of prostate cancer. Thus, this provides the basis that their packaging mechanisms may be cancer-cell specific. In particular, LO-like EVs isolated from prostate-cancer free patients showed they were completely absent of DNA in comparison to patients with prostate cancer, thus suggesting that DNA packaging into LOs is purely a cancer cell-specific mechanism (94). Beyond this, the mechanisms of LO packaging have not been further elucidated. Many studies into LO content have revealed that the containment of certain biomolecules are essential in promoting cancer phenotypes in recipient cells. For instance, the transfer of miR-1227 from LOs to recipient cancer-associated fibroblasts promoted cell migration (98). Additionally, further investigation revealed the enrichment of proteins important for metabolic processes like glucose and glutamine metabolism, which are exceedingly important in cancer progression (3). More recently, the transfer of phosphorylated-Akt1 by LOs into non-cancerous normal human prostate fibroblasts could induce reprogramming to establish tumor-supportive environments (29).

## Harnessing EV packaging for medical uses

### EV cargo loading for therapeutic applications

As described above, EVs are released under vastly different contexts. Thus, the contents of each EV whether it be released from a healthy cell, cancerous cell, or from bacteria can differ greatly. Interestingly, recent studies have unveiled that this variation in cargo harbors strong potential to be used therapeutically. Here we highlight how certain EVs could undergo cargo engineering for the purpose of expanding the repertoire for EV-based therapeutics **(** **Table 2** **).**

**Table 2:** Therapeutic usage of extracellular vesicles.

Therapeutic use	Extracellular vesicle	Summary of use	Clinical stage
Vaccine	Exosome	Exosomes derived from immune cells such as dendritic cells, macrophages have been used to prime immune systems to induce protection against pathogen challenge, specifically against *Mycobacterium tuberculosis* and *Toxoplasma gondii*. Studies into anti-tumor protection using exosomes is also a popular area of study.	Majority of studies are pre-clinical. Phase 2 trial on safety of MSC-derived exosomes recently completed (NCT04313647).
	Microvesicle	MVs derived from cancer cells may provide protection against tumor development. This is thought to be due to the carriage of tumor-specific molecules, such as nucleic acids and proteins.	Majority of studies are pre-clinical. 1 completed Phase 2 study on use of chitin MVs to protect against rhinitis following pollen challenge (NCT00443495).
	Apoptotic bodies	DC-derived ApoBDs can modulate immune cell activation, proliferation, and cytokine release which may provide protection against tumor development. This was also the case for other APC-derived ApoBDs, however the use of ApoBD-based vaccines have not yet been formulated or tested for use in humans beyond this.	All studies are pre-clinical.
	Small apoptotic EVs	Not investigated	Not applicable
	Migrasomes	Not investigated	Not applicable
	Large oncosomes	Not investigated	Not applicable
	Bacterial OMVs	OMVs from bacterial species have been known to induce protection against pathogen challenge. Thus, their use in vaccine development has been paramount for protection against meningococcal B disease as shown through approved use of RmenB-OMV (also known as BEXSERO^®^), an OMV based vaccine with *Neisseria* proteins and OMVs. Furthermore, modification of OMVs to contain other pathogen proteins can also be used to elicit protection, which was the case for Influenza H1N1 and MERS-COV, however these modified OMVs have yet to be further studied in clinical trails. Recent efforts to use OMVs to protect against COVID-19 infection begun.	56 clinical studies completed on BEXSERO^®^, an OMV based vaccine against meningococcal B disease. Has been approved for use. 24 studies (2 phase I, 7 phase 2, 10 phase 3, 3 phase 4) currently investigating its use against STIs in at-risk groups. Alternative OMV-based vaccine strategies are still in pre-clinical stages.
Regenerative medicines	Exosome	MSC-derived exosomes have garnered interest in treatment for cardiovascular, renal, lung and liver pathologies by promoting wound healing through Wnt-B catenin pathways. There is a recent spike in studies investigating therapeutic capacity of MSC-derived EVs to treat COVID-19 associated illnesses.	Most studies are pre-clinical, 2 currently recruiting clinical trials (phase I/II) on MSC-EVs to treat ARDs, T1D (NCT05127122, NCT02138331).
	Microvesicle	Similar to MSC-derived exosomes, MSC-derived MVs have been studied in the context of wound healing and treatment of many different pathologies.	All studies are pre-clinical.
	Apoptotic bodies	Recent studies have shown stem cell derived ApoBDs can promote regeneration of different types of cells including stem cells, epithelial cells, and osteoclasts. This is now becoming more extensively studied, with many groups investigating the role of ApoBDs in regeneration in *in vivo* mouse model or zebrafish systems.	All studies are pre-clinical.
	Small apoptotic EVs	Although the characteristics of small apoptotic EVs have not been well defined, studies have investigated small apoptotic EV usage to treat sepsis, bone and adipocyte formation, wound healing, T1D. It is not fully understood how this occurs, but Fas-dependant mechanism has been implicated.	All studies are pre-clinical.
	Migrasomes	Not investigated	Not applicable
	Large oncosomes	Not investigated	Not applicable
	Bacterial OMVs	Not investigated	Not applicable
Drug delivery	Exosome	Can be loaded with gene therapies and small molecule drugs like curcumin, methotrexate, paclitaxel to deliver therapies whilst evading immune surveillance. Bovine-milk derived exosomes have shown particular promise.	Majority of studies are pre-clinical.
	Microvesicle	Tumor derived and MSC-derived MVs are commonly loaded with chemotherapeutic agents, as well as other drugs in studies to treat a myriad of different conditions. These are also used to circumvent treatment issues due to other characteristics that determine disease severity, like drug resistance.	8 studies in Phase 2. Current clinical trials are investigating drug loading MVs to treat malignant ascities and pleural effusion (NCT02657460, NCT01854866). Studies investigating the role of MVs in COVID-19 infection have been listed to begin recruitment (NCT04448743).
	Apoptotic bodies	Not investigated	Not applicable
	Small apoptotic EVs	Characterisation is still in early stages. Have shown promise as tumor-cell derived apoptotic EVs loaded with methotrexate, doxorubicin, cisplatin or paclitaxel could inhibit tumor growth.	All studies are pre-clinical.
	Migrasomes	Not investigated	Not applicable
	Large oncosomes	Not investigated	Not applicable
	Bacterial OMVs	Not investigated	Not applicable
Diagnostics	Exosome	Exosomes from tumor cells have been analyzed to identify potential miRNA and protein biomarkers for diagnostic purposes. For instance, the ExoDx^®^ exosome gene expression assay has been adapted to allow detection of high-grade prostate cancer markers and lung cancer in exosomes.	53 Studies on exosomes as biomarkers are currently active or recruiting. Majority are observational studies, looking at the differential gene expression levels between patients.
	Microvesicle	Like exosomes, analysis of MVs from tumor cells have been used to identify potential biomarkers. CSF-derived MVs are also currently being investigated as potential biomarkers and indicators of disease severity in Alzheimer’s disease.	18 Active or recruiting studies utilizing MVs as potential biomarkers for different diseases. Majority are observational studies, investigating differential gene expression between patients.
	Apoptotic bodies	Currently the presence of ApoBDs is used to assist with diagnosis of GVHD, SLE, and COVID-19 infection. Lack of specific membrane markers has prevented further use in diagnostics.	All studies are pre-clinical.
	Small apoptotic EVs	Not investigated	Not applicable
	Migrasomes	Not investigated	Not applicable
	Large oncosomes	Large oncosome detection within patient serum can assist with prostate cancer diagnosis. Other markers on LOs, including Cav-1 expression and DIAPH3 deletion can also indicate disease progression.	All studies are pre-clinical.
	Bacterial OMVs	Current studies are investigating the use of OMVs in biofilm formation. A recent study has indicated the detection of OMVs *via* DNA-Aptamers may allow for early detection of bacterial infections.	All studies are pre-clinical.

### EVs in vaccine development

As EVs can carry constituents of cells, EVs can be used to promote immune responses. For instance, the use of EVs in vaccine development has proven to be an effective method of establishing immunoprotective effects. Notably, the use of exosomes in vaccine development has been under investigation for some time as seen through DC-derived exosomes being able to induce protection in mice against *Toxoplasma gondii* oral challenge (99). Additionally, exosomes were also able to induce a protective immune response by triggering an increase in Th1-specific responses due to the packaging of bacterial antigens (25, 99). For macrophage-derived exosomes, similar findings were also observed in studies against *Mycobacterium tuberculosis* challenge in mice (41). Along with the carriage of bacterial antigens, exosomes are able to carry MHC II molecules to assist in antigen presentation, which is a unique method to initiate immune response (39, 41, 100). Furthermore, EVs from dying cells have also displayed potential for vaccine development. For protection against tumor progression, tumor-baring rats vaccinated with monocyte-derived antigen presenting cells that had engulfed ApoBDs had increased survival by 80% (101). Furthermore, DCs that had engulfed ApoBDs derived from leukemic-B cells promoted T cell activation, proliferation and IFN-γ release (102). Although this has not been extensively investigated, this potential could be extended to use other dead cell-derived EVs including necroptotic EVs, where their lysis may aid in the sustained release of tumor-derived and pathogen-derived antigens to mount immune responses. As such, further investigations will need to be conducted to ascertain the true potential of dead cell-derived EVs like ApoBDs and necroptotic EVs in this context.

Notably, bacterial OMVs have exciting potential for use in vaccines against bacterial and viral infections. Along with the carriage of bacterial antigens, OMVs can also induce immunoprotective effects. This is largely due to the fact that they are non-replicative clones of their parental bacteria, yet highly immunogenic (81). OMVs derived from bacterial species like *E. coli, H. pylori*, and *P. gingivalis* were able to induce protection against pathogen challenge by promoting innate and adaptive immune responses as measured by increase IgG titres, and upregulation of pro-inflammatory mediators like NF-κB (81). Interestingly, this could be associated with the selective packaging of major virulence factors from pathogens, like RagA/B from *P. gingivalis (* 82). As mention above, bacteria have displayed selective packaging mechanisms through the inclusion of virulence factors and simultaneous exclusion of membrane proteins in OMVs (82). This provides the potential for bacterial OMVs to be engineered to contain pathogen-derived cargo for vaccine development. Although this has yet to be investigated extensively, OMVs have been engineered to display pathogen-derived antigens on their surface. *E.coli* derived OMVs were engineered to display M2e (an Influenza A virus matrix protein) on the surface of OMVs, which induced effective immunoprotection against influenza A virus when used to vaccinate BALB/C mice (103). Of note, OMV-based vaccines have also displayed promise in providing protection against viruses like Influenza strain H1N1 and MERS-CoV, and SARS-CoV-2 (104). Importantly, OMV-based vaccine RMenB-OMV (also known as BEXSERO^®^) has been approved for protection against meningococcal B disease, and is currently under investigation for protection against STIs like Gonorrhoea and HIV (NCT04415424, NCT04597424) (105) **(** **Table 2** **)**. A recent intranasal vaccine candidate based off *Salmonella typhimurium* OMVs has also been investigated. These OMVs are modified with SARS-CoV-2 spike receptor binding domains, and successfully created neutralizing antibody responses in vaccinated participants exposed to wild-type and delta variants, thus expanding the world’s vaccine repertoire to protect against COVID-19 variants of concern (33).

### EVs in regenerative medicine

An interesting development of EV-based therapies is their use in regenerative medicines. For instance, the use of mesenchymal stem cell (MSC)-derived EVs has garnered recent interest as they have been used to treat many diseases. This includes cardiovascular, renal, lung and liver pathologies (16, 18, 106). Notably, MSC-derived EVs are also known to promote wound healing as shown in a rat skin burn model. Interestingly, these healing properties were attributed to the delivery of Wnt4 molecules by MSC-exosomes, thus activating Wnt/β-catenin signaling to induce skin cell proliferation and migration (107). MSC-derived ApoBDs were also able to rescue stem cells through Wnt pathway activation through the transferral of E3 ligase RNF146 and miR-328-3p (108). As an alternative mechanism, recently small EVs generated from apoptotic MSCs were found to promote bone and adipocyte formation following engulfment in MRL/lpr and Casp3^-/-^ mice by upregulating pro-angiogenic genes THSN1 and VASH1 (109). Although this study did not investigate the contents of the small apoptotic EVs, it suggests that like other MSC-derived EVs, EVs of this origin could contain specific cargo to assist in the proliferation of many cell types.

Interestingly, ApoBDs derived from cells besides stem cells are also able to promote cell proliferation. Again through Wnt signaling, ApoBDs derived from zebrafish epithelial cells were able to promote stem cell proliferation through caspase-dependent packaging of Wnt8a molecules (24). Furthermore, ApoBDs from osteoclasts could also induce osteoclastogenesis and differentiation through RANK-L packaging (110, 111). These studies highlight the potential that ApoBDs could be specifically engineered or harnessed for use in the regeneration and proliferation in different pathologies. Other dead cell-derived EVs like necroptotic EVs could also be used for regenerative medicine, as recent evidence suggests continued synthesis of cytokines following lysis of necrotic cell ‘corpses’ could be harnessed to contain new cargo to promote proliferation (2, 112). Although promising, further studies will need to be conducted in assessing the feasibility and efficacy of these EV-based treatments.

### EVs in drug delivery

As there are a myriad of ways to deliver therapeutics, EVs are currently being explored as promising drug delivery vehicles. There are a number of benefits in utilizing EVs as a drug delivery platform, including their size, similarity to parental cells, and ability to avoid immunosurveillance mechanisms (113). Notably, using EVs to deliver small molecule drugs may provide a way to enhance drug bioavailability by avoiding increased drug dosages, which can be detrimental to patients. Furthermore, there is a potential to deliver gene therapies *via* EVs as well. Exosomes and MVs in particular have been well-characterized in the delivery of many different molecules including nucleic acids and small molecule drugs (36). For instance, exosomes could be loaded with siRNA to induce significant knockdown of beta-secretase 1 in mice, providing gene-based therapies for Alzheimer’s disease (114). Furthermore, the packaging of small molecule drug paclitaxel (PTX) into exosomes and MVs was able to increase cytotoxicity against PC3 and LNCaP cells (115). Interestingly, macrophage-derived exosomes packaged with PTX was able to reduce the size of tumors in mice with pulmonary metastases in comparison to mice treated with the drug Taxol only (116). This was also reflected in bovine milk-derived exosomes also packaged with PTX and other drugs like withaferin A, as they displayed higher anti-cancer, anti-inflammatory effects, and increased bioavailability of the drugs *in vivo (* 113). Furthermore, packaging of MVs with MTX or cisplatin were able to reverse drug-resistant properties in tumor cells from patients with end-stage lung cancer, as well as cholangiocarcinomas (117, 118). As such, the therapeutic effects of EV use in drug loading has progressed such that the loading of molecules like curcumin into exosomes and MVs is currently under investigation in clinical trials (119, 120) **(** **Table 2** **)**.

Besides exosomes and MVs, other EV subtypes also exhibit drug delivery properties. For example, migrasomes are also able to transport cytosolic contents and organelles as indicated by the quick transfer of TSPAN4-GFP along retraction fibres during biogenesis, and movement of damaged mitochondria (4, 90, 92). The recent discovery of ROCK1 regulating migrasome formation also provides an avenue for their formation to be exploited therapeutically (91). Although these avenues have not been further investigated due to the early stages of migrasome research, it suggests another potential avenue for drug therapies to be transferred to recipient cells. Additionally, other dead cell-derived EVs have displayed promise through their ability to contain and transfer important bioactive molecules (60, 62, 63, 121, 122). Apoptotic tumor-derived EVs loaded with methotrexate (MTX), doxorubicin, cisplatin or PTX were able to inhibit tumor growth *in vivo* without adverse side-effects (123). Providing further demonstration for the use of dead-cell derived vesicles in therapeutics, promising work has shown apoptotic vesicles can be loaded with either anti-inflammatory molecules like curcumin, or various pro-drugs in order to provide benefit (12, 28, 122). In the case of the latter, the loading of apoptotic vesicles with disulphide-linked drugs, camptothecin and PR104A (anti-cancer agents) could effectively promote drug penetration in whole tumors promoting tumor destruction (122). Nevertheless, there are still many avenues to explore in determining the best use of EVs in drug delivery, including development of mass-production methods and quality control (113).

### EVs in diagnostics

EVs are shed by virtually all cells under normal and pathological conditions. Importantly, because they carry protein and nucleic acids that reflect their parental cell origin, they are thought to provide the key in early detection of various diseases. For instance, numerous studies have indicated that analysis of miRNAs packaged into urinary EVs from prostate cancer patients can assist with early diagnosis (32, 124, 125). This has also extended to include cardiovascular diseases, pathogen-specific conditions, neurological diseases, and traumatic brain injuries (126–128). Additionally, because EVs are widely distributed in biological fluids, they are more readily attainable through liquid biopsies using blood, urine, saliva, sperm, or breast milk (36, 113). This has already provided vast advancements within the field, as the use of EV-based tests in conjunction with mainstay diagnostic techniques can increase test specificity. This was exemplified through combination of ExoDx^®^ *Prostate(IntelliScore)* (a urine exosome gene expression assay, Bio-techne) and prostate serum antigen (PSA) testing to detect high-grade prostate cancer prior to tissue biopsies (32, 124). This assay has also been adapted to detect lung cancer markers, known as ExoDx^®^ *Lung(ALK) (* 129) **(** **Table 2** **)**.

As mentioned above, it is currently well known that analysis of miRNAs packaged in EVs can assist with classification of cancers and understanding their complexities (94). This was particularly important for unveiling therapeutic resistance in ovarian tumors, which was indicated by specific miRNA enrichment (130). Likewise, analysis of miRNA in semen-derived exosomes demonstrated the overexpression of miR-142-3p, miR-142-5p, and miR-223-3p in malignant and benign prostate tumors from patients, in comparison with healthy controls (124). Aside from miRNAs, other proteins like cancer-specific biomarkers have been located within MVs to assist with diagnosis of specific cancers. For instance, colon cancer biomarkers CEACAM1 and MUC13 could be found in MVs derived from colon cancer cells, which could assist in the diagnoses of colorectal cancers (131). Similarly, protein tyrosine kinase 2 (PTK2) (which indicates oncogenic transformation) could also be tracked in MVs derived from MDAMB231 cells, further suggesting that analysis of EV content is an attractive target for diagnostics in breast cancer (132). This is also the case for other pathological conditions such as Alzheimer’s disease. Notably, cerebrospinal fluid-derived MVs from Alzheimer’s patients displayed reduced concentration of tau and APP, which can indicate disease severity and associated cognitive decline (133).

LOs can also be useful in diagnostics as they can be detected in the bloodstream of mice and patients with prostate cancer (3). For instance, expression of Cav-1 in LOs is known to be an indicator of metastatic disease in prostate cancer patients (98). Additionally, the deletion of DIAPH3, which is important in LO biogenesis, may also be an indicator of metastasis as its deletion was detected in 64% of metastatic tumors (95). Furthermore, as LOs are known to carry oncoproteins like MyrAkt1 and metalloproteinases (which are important in maintaining tumor microenvironments), their detection could further assist with the diagnosis and monitoring of other cancers (97).

Additionally, as a variety of diseases can be associated with death of specific cell types, the analysis of dead-cell EVs could be used to monitor the level of cell death of a particular cell origin. For instance, cell-type specific ApoBDs can be isolated from biological tissues by using cell-type specific markers, which can potentially aid diagnosis of different conditions (69). Furthermore, the analysis of EV content may also assist in diagnosis, as analysis of apoptotic EVs revealed packaging of spliceosomes, which is important in identifying glioblastoma (63, 134). For other conditions like graft-vs-host-disease (GVHD), the presence of ApoBDs within the crypts of gastrointestinal tracts can aid in diagnosis. However, because further investigation is required to diagnose GVHD, analysis of ApoBD content could mitigate this step and create a quicker and easier process of diagnosis (30). Likewise, the detection and isolation of bacterial OMVs may also assist with early diagnosis and prevention of bacterial infection. The advent of a DNA-aptamer shown to be highly sensitive to bacterial OMVs may allow this (135). Although many advances have been made to improve on diagnostic techniques, the use of EV cargo analysis requires further investigation. Advancements into EV isolation techniques, characterisation, detection, and safety under different pathological conditions is needed to further advance diagnostic outcomes.

## Concluding remarks and future perspectives

Over the years, many advances have been made in better understanding the mechanisms underpinning the importance of EV packaging pathways. The subtle, yet important differences within biomolecule carriage and transfer mechanisms among traditional EV subtypes vs newly discovered EVs have highlighted. Their potential to be utilized in providing therapeutic benefit for many conditions. In brief, it is known exosomes, MVs and ApoBDs can contain and package specific cargo through protein-specific pathways. Recently emerging research has shown unique packaging mechanisms harnessed by OMVs, LOs and migrasomes related to their biogenesis. They can import cellular material through new avenues, providing new protein targets, insights, and avenues for researchers to utilize and ultimately advance EV-based research. In this review, we have discussed how these characteristics could be exploited therapeutically, in vaccine development, regenerative medicines, drug delivery and lastly within diagnostic studies. Furthermore, we have highlighted how various groups have harnessed these bioengineering aspects to enhance characteristics of EVs to provide more suitable treatment options for different pathologies. This includes drug loading and expressing viral proteins for vaccine development, among others. It is no doubt that each EV subtype discussed in this review could be utilized in treatments, as there is no “one-size-fits-all” option. Overall, additional insight into the content and packaging mechanisms of less studied EV subtypes may hold the key to advancing modern-EV based therapeutics and creating a diverse library of options available for the treatment of various pathophysiological conditions.

## Author contributions

All authors listed have made a substantial, direct, and intellectual contribution to the work and approved it for publication.

## Funding

This work was funded by the National Health and Medical Research Council [GNT1125033, GNT1140187].

## Conflict of interest

The authors declare that the research was conducted in the absence of any commercial or financial relationships that could be construed as a potential conflict of interest.

## Publisher’s note

All claims expressed in this article are solely those of the authors and do not necessarily represent those of their affiliated organizations, or those of the publisher, the editors and the reviewers. Any product that may be evaluated in this article, or claim that may be made by its manufacturer, is not guaranteed or endorsed by the publisher.
